# The Impact of Secondary Particles on the Association between Ambient Ozone and Mortality

**DOI:** 10.1289/ehp.10777

**Published:** 2008-01-10

**Authors:** Meredith Franklin, Joel Schwartz

**Affiliations:** Department of Environmental Health, Harvard School of Public Health, Boston, Massachusetts, USA

**Keywords:** confounding, meta-analysis, mortality, nitrate, organic carbon, ozone, PM_2.5_, secondary pollutants, sulfates, time series

## Abstract

**Background:**

Although several previous studies have found a positive association between ambient ozone and mortality, the observed effect may be confounded by other secondary pollutants that are produced concurrently with ozone.

**Objectives:**

We addressed the question of whether the ozone–mortality relationship is entirely a reflection of the adverse effect of ozone, or whether it is, at least in part, an effect of other secondary pollutants.

**Methods:**

Separate time-series models were fit to 3–6 years of data between 2000 and 2005 from 18 U.S. communities. The association between nonaccidental mortality was examined with ozone alone and with ozone after adjustment for fine particle mass, sulfate, organic carbon, or nitrate concentrations. The effect estimates from each of these models were pooled using a random-effects meta-analysis to obtain an across-community average.

**Results:**

We found a 0.89% [95% confidence interval (CI), 0.45–1.33%] increase in nonaccidental mortality with a 10-ppb increase in same-day 24-hr summertime ozone across the 18 communities. After adjustment for PM_2.5_ (particulate matter with aerodynamic diameter ≤ 2.5 μm) mass or nitrate, this estimate decreased slightly; but when adjusted for particle sulfate, the estimate was substantially reduced to 0.58% (95% CI, –0.33 to 1.49%).

**Conclusions:**

Our results demonstrate that the association between ozone and mortality is confounded by particle sulfate, suggesting that some secondary particle pollutants could be responsible for part of the observed ozone effect.

Tropospheric ozone, a secondary pollutant, is a major constituent of photochemical smog. There is very strong scientific evidence that exposure to ozone leads to a variety of adverse health outcomes. Recent population-based time-series studies (Bell et al. 2004; Gryparis et al. 2004) and meta-analyses (Bell et al. 2005; Ito et al. 2005; Levy et al. 2005) have shown that there is an increased risk of mortality associated with short-term exposure to ozone air pollution. Photochemical smog is produced in large part by the interaction of combustion emissions with sunlight and is exacerbated by warm temperatures. Consequently, high ozone episodes are typically observed on sunny, hot days. In assessing ozone-related health effects, it is therefore very important to control for the confounding effect of temperature. This has become common practice in time-series analyses of ozone, and a significant amount of attention has been paid to determining the most appropriate model specification of temperature (Basu and Samet 2002; Ito et al. 2005; Schwartz 2004; Thurston and Ito 2001).

Interpretation of ozone–mortality data is also complicated by the potential for ozone effects to be confounded by other pollutants. Sulfate, organic particles, and nitrate—all secondary pollutants resulting largely from combustion sources—are often produced concurrently and by photochemistry similar to that of ozone. It is possible that an observed relationship between ozone and mortality is really capturing an effect between other secondary aerosols and mortality which has not been accounted for in the model. Although it has been shown that PM_10_ (particulate matter with an aerodynamic diameter ≤ 10 μm) does not confound the ozone association (Bell et al. 2004), this is likely attributable to the fact that PM_10_ originates from different sources than ozone, and it does not typically co-vary with ozone. This is not the case for PM_2.5_ (PM with an aerodynamic diameter ≤ 2.5μm). Total PM_2.5_ mass represents the combination of all secondary particles as well as primary particles, but is more specific to combustion-related particles than PM_10_. Short-term exposure to PM_2.5_ has been associated with increased mortality (Franklin et al. 2007). Components of PM_2.5_ that are mostly secondary in nature have not been extensively examined in an epidemiologic setting largely because of the lack of reliable PM_2.5_ mass and species data. The U.S. Environmental Protection Agency’s (U.S. EPA 2007) routine measurement of PM_2.5_ chemical components began around 2000–2001. The time series of these data are further limited by the fact that concentrations are collected only every third or sixth day.

In this study, we addressed the question of whether the ozone–mortality relationship is entirely a reflection of the adverse effect of ozone or whether it is, at least in part, an effect of other secondary pollutants. We conducted a time-series analysis for 18 U.S. communities in which the effect of ozone was examined alone and subsequently after including PM_2.5_, particle sulfate, organic carbon (OC), and nitrate separately in the model. We then pooled the community-specific ozone–mortality effect estimates using a random-effects meta-analysis to obtain an overall population effect.

## Materials and Methods

### Data

We selected 18 communities on the basis that ozone, PM_2.5_ mass, PM_2.5_ speciation, and daily mortality were available for at least 3 years between 2000 and 2005. The ozone, PM_2.5_ mass, and PM_2.5_ speciation data were obtained online from the U.S. EPA Technology Transfer Network Air Quality System (U.S. EPA 2007), and daily mortality records were obtained from both the National Center for Health Statistics (NCHS; Hyattsville, MD) and various state departments of health. Meteorologic data including temperature and dew point temperature, necessary to control for confounding in the ozone–mortality relationship, were acquired from the National Climatic Data Center (NCDC 2007).

Because the NCHS no longer provided the date of death in their national mortality record data sets after 2000, it was necessary to acquire data through requests directly to state public health departments (California, Massachusetts, Michigan, Minnesota, Missouri, New York, Ohio, Pennsylvania, Texas, Washington). The analysis was conducted at the county level because this was the smallest resolution available for all mortality data. Communities were defined as a single county or contiguous counties, and they were named by the major city located within it. The mortality data used provide nonconfidential information on decedents including state of death, county of death, age, sex, date of death, and primary cause of death. Only those individuals who died of nonaccidental causes were examined [i.e., *International Classification of Diseases, 10th Revision* codes V01 through Y98 (World Health Organization 1993) were excluded].

Because ozone is chiefly a concern in the warmer months, many cities monitor it only in the warm season. Moreover, the association between ozone and mortality seems to be restricted to the warm season (Levy et al. 2005; Schwartz 2004). Hence, we conducted the analysis for the 5 months from May to September.

Ozone monitors operate on an hourly basis, whereas the PM_2.5_ monitors used in this analysis operate on a 24-hr schedule. For comparability purposes, daily average ozone concentrations were used.

There are typically multiple ozone and PM_2.5_ mass monitors within a community. To make use of all information available from the monitoring sites within each of the 18 communities studied, we averaged the daily ozone and mass concentrations over the community using a method adapted from Schwartz (2000). This averaging method accounts for the fact that monitors do not all report concentrations on the same days, resulting in an imbalance in the daily data used to take the community-wide average. However, before averaging, any monitor that was not well correlated with the others (*r* < 0.8 for two or more monitor pairs within a community) was excluded because it likely measured a local pollution source and would not represent the general population exposure over the entire community. There is typically only one speciation trends network (STN) monitor within a community so data from a single site were used to characterize sulfate, OC, and nitrate. These monitors only operate on a 1-in-3 or 1-in-6 day cycle. The OC data were not blank corrected and thus had a positive bias due to sampling artifacts. Blank corrections for these monitors were not made available until 2003, and these data had many missing values. To determine a suitable correction, a monthly average of the available blanks was taken for each monitor. These correction factors were then subtracted from the reported OC values over the whole study period.

### Statistical methods

Community-specific daily mortality counts were matched by day with daily ozone and PM_2.5_ mass concentrations, sulfate, OC, nitrate, temperature, and dew point temperature. In the first step, we performed a time-series analysis on the matched data set for each community using Poisson regression. We used separate models to examine the effect of ozone alone and ozone adjusted for each of the secondary particle pollutants. The secondary particle confounders were each included as linear terms in the model, but we also examined the possibility that the confounding effect of PM_2.5_ on ozone might not be linear by including a cubic regression spline for PM_2.5_.

Three different exposure lags of ozone (lag0, lag1, and 2-day averaged lag01) were examined. Previous studies found the strongest association with ozone at lag0 and lag01 (Bell et al. 2004; Levy et al. 2005).

Because we were analyzing data only from the warm season, the confounding effects of temperature and dew point temperature were controlled for by taking their 3-day running mean and including them in the Poisson model as linear terms. The association between mortality and temperature is often strongest over longer exposure periods (Braga et al. 2002). Although nonlinear terms of temperature have been used to examine its association with mortality in full year models, linear terms have been sufficient when examining just the warm season (Basu and Samet 2002). Furthermore, a recent case–crossover study concluded that matching control days on temperature did not change the ozone effect compared with models that used parametric control for temperature (Schwartz 2004). This suggested that the possibility of confounding by temperature on the day of death was unlikely.

We tested our *a priori* specification of temperature by conducting a sensitivity analysis of the ozone effect under different temperature parameterizations. We examined temperature and dew point temperature as cubic regression splines with 3 degrees of freedom (df), combinations of same-day and running means, quadratic terms, and removing the hottest 2% of days.

Day of the week was controlled for with indicator variables and time was controlled for with a cubic regression spline with 3 degrees of freedom for each 5-month warm season.

Because the PM_2.5_ mass and species concentrations were not available daily, we estimated the ozone–mortality effect for various subsets of the data. For example, to examine whether sulfate was a confounder, we computed the association between ozone and mortality restricted to the days where sulfate concentrations were available. Then we included sulfate in the model and reexamined the ozone–mortality effect estimate. This allowed for better assessment of the degree of confounding by the secondary species. We reduced the number of degrees of freedom used in the cubic regression spline for time to reflect the percentage of total days available for the model.

We combined the effect estimates and standard errors from the community-specific Poisson regression models using random-effects meta-analysis to obtain an overall effect across the study domain (Berkey et al. 1998). The results are expressed as the percent increase in nonaccidental mortality with a 10-ppb increase in 24-hr summertime ozone concentration.

## Results

Summary statistics of the air pollution and mortality data used for each of the communities are shown in Table 1. There was more than a two-fold difference in the average ozone concentrations over the 18 communities examined. Concentrations were highest in Fresno (48.7 ppb) and Riverside (45.1 ppb), California, and were lowest in Seattle, Washington (21.4 ppb). The summertime averages of PM_2.5_ were highest in Riverside (27.9 μg/m^3^) and Pittsburgh, Pennsylvania (18.4 μg/m^3^). Although these concentrations were well above the National Ambient Air Quality Standard (NAAQS) of 15 μg/m^3^, only 7 of the 18 communities examined exceeded the standard (U.S. EPA 1997). Most eastern U.S. communities had summer peaking PM_2.5_ concentrations, but this was not necessarily true for communities in the central or western areas. Because of the 1-in-3 or 1-in-6 day sampling schedule on which the STN monitors operate, there were substantially smaller numbers of sulfate and OC concentrations available than for ozone and PM_2.5_ mass data. On average, sulfate concentrations were higher in the East than West, with Pittsburgh having the highest concentration (8.8μg/m^3^). Sulfate accounted for 29–48% of the mass in the East, 17–35% in the Midwest and South, and 17–33% in the West. Organic carbon was highest in Riverside (6.4 μg/m^3^), which represented 42% of the total mass. In other communities such as Fresno, California (4.7 μg/m^3^), Cleveland, Ohio (4.5 μg/m^3^), and Pittsburgh (4.5 μg/m^3^), which had high OC levels, they only represented 23–25% of the total mass. Sacramento, California, had low OC (3.3 μg/m^3^) but this was 40% of the total mass. OC accounted for only 17–28% in the other communities. Nitrate concentrations were highest and accounted for almost 50% of the PM_2.5_ mass in Riverside. In other California communities it accounted for < 30% of the mass; in the remaining communities it accounted for < 11% of the mass. The average temperatures did not vary widely across the communities because these data represented summer months only.

**Figure 1:**
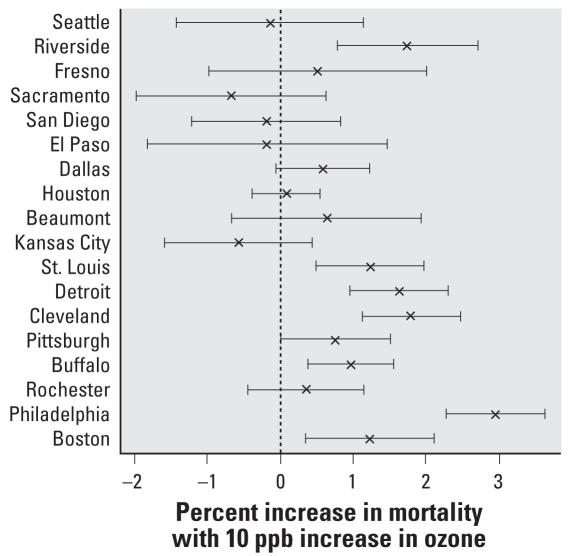
Community-specific estimates (with 95% confidence intervals) for the percent increase in nonaccidental mortality per 10-ppb increase in same-day 24-hr summertime ozone concentrations

The community-specific estimates of the association between lag0 24-hr summertime ozone and nonaccidental mortality obtained from the separate Poisson regression models are shown in Figure 1.

The meta-analysis results of testing different temperature parameterizations are presented in Table 2. By including a cubic regression spline (with 3 df) for the 3-day running mean of temperature and dew point temperature, the lag0 ozone effect increased slightly to 0.93% [95% confidence interval (CI), 0.50–1.35%]. Including linear terms for the 3-day running mean of temperature and dew point temperature, as well as separate terms for unaveraged temperature and dew point temperature, resulted in a slightly decreased effect estimate [0.79% (95% CI, 0.40–1.18%)]. A similar result was found after removing the hottest 2% of days in each of the communities and fitting the model with 3-day running mean of temperature and dew point temperature [0.78% (95% CI, 0.26–1.29%)]. A large decrease in the effect size occurred when the models included just a linear term for same-day temperature and dew point temperature [0.48% (95% CI, 0.05–0.90%)] or as quadratic terms [0.45% (95% CI, 0.004–0.89%)]. This decrease may be explained by the fact that same-day temperature alone does not likely control for longer-term confounding. Schwartz (2004) found that the ozone effect was not confounded by same-day temperature.

The difference in the magnitude of the ozone effect between the model controlling for temperature and dew point temperature with only two linear 3-day running means and the models with either two cubic regression splines or four linear terms was small. Therefore, we chose the more parsimonious model out of concern that the sample size was greatly reduced once we included sulfate, OC, and nitrate.

Ozone was moderately correlated with PM_2.5_, sulfate, OC, and nitrate, as shown in Table 3. The meta-analysis results for before and after adjustment for PM_2.5_, sulfate, OC, and nitrate are shown in Table 4. For a 10-ppb increase in lag0 24-hr summertime ozone concentration, there was a 0.89% (95% CI, 0.45–1.33%) increase in nonaccidental deaths. The effect at lag01 was similar [0.87% (95% CI, 0.25–1.50%)], but the effect at lag1 was much smaller and not statistically significant [0.33% (95% CI, –0.28 to 0.94%)]. Because the particle speciation data were sparse, it was not feasible to look at their 2-day average lag effect. Furthermore, three of the communities had PM_2.5_ mass concentrations that were, even after averaging over multiple sites, for every third day. Thus we used the lag0 ozone effect estimate to assess confounding.

Only 84% of the full data set was available when we restricted the analysis to days where PM_2.5_ mass concentrations were available. The effect estimate of ozone on these days was almost unchanged [0.88% (95% CI, 0.39–1.36%)]. After adjustment for PM_2.5_ both as a linear term and as a cubic regression spline, the ozone effect was only slightly reduced. This suggested that PM_2.5_ was not a strong confounder.

**Figure 2:**
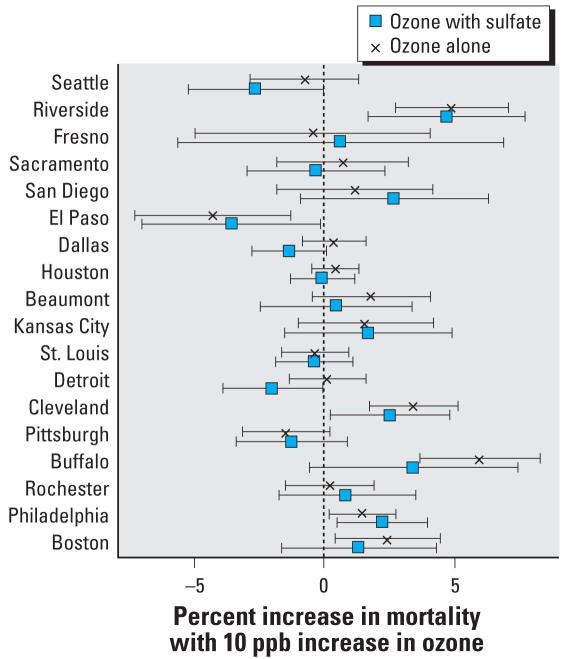
Community-specific estimates (with 95% CI) for the percent increase in nonaccidental mortality per 10-ppb increase in same-day 24-hr summertime ozone concentration before and after adjustment for particle sulfate.

When restricted to days where particle species concentrations were available, the data set shrank even further to 18% for sulfate, 17% for OC, and 17% for nitrate. Of these secondary particle species, only sulfate demonstrated substantial confounding as the ozone effect estimate decreased from 0.85% (95% CI, –0.01 to 1.55%) to 0.58% (95% CI, –0.33 to 1.49%). The community-specific estimates of the association between ozone and nonaccidental mortality before and after adjustment for sulfate are shown in Figure 2. There was a modest decrease in the ozone effect after adjustment for nitrate (0.12%). The fact that fewer observations were available to model the association between ozone and mortality after adjustment for secondary particles is a major factor for the wider confidence intervals for those estimates.

As a final sensitivity analysis, we took the predicted seasonal pattern from the “full” ozone model, which included all days, and used it as an offset in the reduced models. This was a control for the seasonal pattern rather than using cubic splines for time. The results were very similar to those reported in Table 4 (data not shown).

To assess whether the moderate correlations between ozone and the secondary particle pollutants had any effect on the model, we examined the correlation between the estimated ozone and particle coefficients in each of the communities. There were 10 correlations between ozone and PM_2.5_ which were in the range of –0.20 to –0.28. The strongest correlation between sulfate and ozone was in Philadelphia (0.33), but in most communities was only between –0.02 and 0.1. Similarly low correlations were found between the ozone coefficient and both OC and nitrate.

## Discussion

In this multicommunity study, we confirmed the previously reported association between ozone and daily deaths. After pooling 18 community-specific effect estimates, we found that over our study population there was a 0.89% (95% CI, 0.45–1.33%) increase in daily deaths for a 10-ppb increase in 24-hr summertime ozone. This effect size is almost identical to that reported in a meta-analysis conducted by Bell et al. (2005) of 39 separate ozone time-series studies. They also found that the ozone effect estimate was not different when the meta-analysis was stratified by whether or not the original study had adjusted for PM (either PM_2.5_ or PM_10_ but they were not distinguished). Similarly, in the National Morbidity, Mortality, and Air Pollution Study (Bell et al. 2004), adjustment for PM_10_ did not affect the overall ozone–mortality effect estimate, and a more recent examination of these data (Bell et al. 2007b) showed that PM_2.5_ also did not alter the effect. Although these have been valuable findings, these studies did not examine the more relevant issue of confounding by secondary particles which, because of photo-chemical formation processes similar to those of ozone, are more likely confounders of the ozone–mortality effect.

We first addressed the possibility that the ozone–mortality association could be more strongly confounded by fine particle concentration than by coarse particles, because fine particles are associated with combustion sources and are composed largely of secondary products. However, after including PM_2.5_ mass in the model we found that the ozone–mortality association was essentially unchanged. This result, which agrees with Bell et al. (2007b), strengthens the argument for a causal association. Yet when we controlled for specific secondary particles that are also partly the result of photochemistry, the situation changed. Although it would be expected that the small sample size resulting from infrequent STN network measurements could reduce the precision, and subsequently the significance of the effect estimate, it should not bias the effect size. We found substantial attenuation of the coefficient for ozone with control for sulfate, from 0.85% to 0.58%, a percent decrease of 32%. Significantly, the unadjusted ozone effect estimate was similar to the full model effect even when restricted to the days where sulfate concentrations were available (18% of full data set), and it was almost statistically significant at the 0.05 level.

It is difficult to draw any conclusions with respect to the confounding effect of OC. The odd behavior of the ozone effect estimate restricted to available OC days could be a result of having a biased sample. There were fewer OC observations than sulfate observations, and the missing OC observations (where there were not missing sulfate observations) were mostly in Rochester, New York, and St. Louis, Missouri. The blank correction reduced the number of observations in Rochester because several low OC concentrations became negative and were subsequently removed from the analysis. On the other hand, the missing observations in St. Louis were likely attributable to an unreported instrument issue. A sensitivity analysis was conducted and we found that using OC concentrations that were not blank corrected resulted in similar effect sizes. However, when we removed St. Louis from the analysis, the ozone effect estimate increased to 0.71% (95% CI, –0.24 to 1.66%) before adjustment, and after adjustment for blank-corrected OC it remained almost unchanged [0.70% (95% CI, –0.26 to 1.65%)]. This suggests that there is little confounding of the ozone–mortality association by OC.

Epidemiologic evidence of an association between sulfate particles and daily deaths has been found previously (Goldberg et al. 2001; Maynard et al. 2007). More recent studies provide biological plausibility for the association as they have shown an association between sulfate concentrations and arrhythmias (Sarnat et al. 2006), reduced heart rate variability (Chuang et al. 2007a, 2007b; Luttmann-Gibson et al. 2006), increased markers of inflammation, thrombosis, and oxidative stress in the blood (Chuang et al. 2007a; O’Neill et al. 2007), and reduced brachial artery reactivity (O’Neill et al. 2005). Furthermore, studies of the association between personal exposures and ambient concentrations of various pollutants have indicated that day-to-day changes in ambient ozone may be a better predictor of day-to-day changes in personal exposure to sulfate than in exposure to ozone itself (Sarnat et al. 2001, 2005).

It is not too surprising, even with limited statistical power, that we did not detect any confounding effect of OC or nitrate. Little is known about the health effects associated with OC, and in a recent review, Schlesinger (2007) indicated that little epidemiologic or toxicologic evidence exists of adverse health effects associated with current nitrate levels.

Although the estimated impact of ozone on mortality was reduced by controlling for sulfate, it did not disappear. Ozone is known to produce biological responses including respiratory inflammation, which may inhibit recovery from infection, or indirectly result in systemic effects. For example, a recent panel study using personal air monitors on students living on a campus found that ozone was associated with increase levels of C-reactive protein, fibrinogen, 8-hydroxy-2′-deoxyguanosine, plasminogen activator inhibitor-1, and decreased heart rate variability (Chuang et al. 2007a). Ozone exposure was also shown to lead to changes in heart rate and decreased heart rate variability in 21 Boston adults (Gold et al. 2000). However, these outcomes were more strongly associated with PM_2.5_.

There are a number of limitations to this analysis. An obvious one is the sparseness of the STN data, giving us lower power than we would desire. There are other limitations with respect to this monitoring network. Sulfate, OC, and nitrate particles are not the only secondary pollutants. There are other photo-chemical gases including organic compounds such as aldehydes and peroxyacetyl nitrate, but we were unable to examine the extent to which ozone stands for them because they are not measured routinely. It is also puzzling that control for PM_2.5_ had essentially no impact on the ozone coefficient, particularly because secondary particles are a major component of PM_2.5_ in the warm season. Our analysis was limited to 18 communities that were not necessarily representative of the entire United States. Although there were five communities on the West coast, six in the Midwest and South, and seven in the East, many of these communities were clustered together in particular states because of the availability of mortality data that we requested from various state departments of health. Given the geographic variability in the chemical composition of PM_2.5_, the location of the communities examined could have an impact on our results. For instance, with sulfate concentrations being consistently higher east of the Ohio River (Bell et al. 2007a), our estimate of its confounding effect might have been different (likely stronger) had we been able to include more communities from this region. Furthermore, we were also limited to examining effects in the summer because this is the only time the U.S. EPA’s ozone network collects nationwide data. Although ozone concentrations typically peak in the summer in all communities, this is not generally true for all particle species, particularly nitrate. A seasonal analysis would shed light on whether nitrate has a greater confounding of the ozone–mortality association in the winter.

Collinearity presents a major problem in epidemiologic studies, and in combination with differential amounts of covariate measurement error, interpretation of the coefficients can be difficult. It is known that when covariates A and B are highly correlated, the standard errors of the estimated coefficients will be inflated. It is similarly known that if A and B are not correlated but possess measurement error, the effect of the measurement error would be to decrease the estimated effect sizes. However, when correlation and measurement error both exist, the interpretation of the estimated coefficients of A and B becomes clouded. For example, in the most severe case, one might conclude that there is an association between the outcome Y and covariate A while controlling for B, even though the association between Y and A is spurious and the true association is between Y and B. Nevertheless, as noted by Zeger et al. (2000), such a scenario is quite unlikely because it is hard to achieve the combination of high degree of measurement error and high degree of correlation between covariates this would require. In our case, the moderate correlations between ozone and the other pollutants are inconsistent with strong shifting of effects from ozone to secondary particles, based on their simulations. However, some degree of shifting may have occurred if the ozone measurement error is large enough. The simple test of correlation between the estimated ozone and particle coefficients indicated that collinearity was likely not an issue in our analysis.

In summary, our results showed that some of the excess mortality associated with ambient ozone could actually represent the effects of secondary sulfate particles. As in previous studies, confounding of the ozone– mortality association by PM_2.5_ mass was not detected. Additional analyses with a more complete time series of particle speciation concentrations would reduce the uncertainty in our findings. Further study into the issue of confounding by other secondary species is also warranted.

## Correction

In Table 4, the righthand column (“Decrease in effect”), which appeared in the manuscript originally published online, has been removed.

## Figures and Tables

**Table 1:** Summary statistics for 18 U.S. communities.

Community^a^	Years of data^b^	Days of ozone (no.)	Days of PM_2.5_ speciation (no.)	Average daily ozone^c^ (ppb)	Average daily PM_2.5_^c^ (μg/m^3^)	Average daily sulfate (μg/m^3^)	Average daily OC (μg/m^3^)	Average daily nitrate (μg/m^3^)	Average daily temp (°C)	Deaths analyzed ozone matching (no.)	Deaths analyzed ozone + speciation matching (no.)
Seattle, WA	2000–4	765	236	21.4 ± 7.1	7.8 ± 3.5	1.5 ± 0.7	2.2 ± 1.2	0.6 ± 0.3	16.5 ± 3.5	23,568	7,318
Sacramento, CA	2000–3	612	162	34.6 ± 7.9	8.3 ± 3.8	1.5 ± 0.8	3.3 ± 2.2	0.9 ± 0.6	22.5 ± 3.5	14,697	3,605
Fresno, CA	2000–3	612	132	48.7 ± 8.3	11.2 ± 5.1	1.9 ± 0.7	4.7 ± 2.5	1.8 ± 1.2	25.9 ± 3.9	8,549	1,242
Riverside, CA	2001–3	612	116	45.1 ± 9.5	27.9 ± 12.6	4.7 ± 1.8	6.4 ± 2.1	13.4 ± 6.5	22.4 ± 3.5	17,747	3,288
San Diego, CA	2001–3	1,220	152	33.0 ± 7.1	13.3 ± 4.6	4.4 ± 1.9	3.3 ± 1.5	3.6 ± 1.9	20.0 ± 2.1	59,798	7,142
El Paso, TX	2001–5	909	287	29.1 ± 11.6	13.1 ± 7.6	4.1 ± 2.7	2.5 ± 2.0	0.4 ± 0.2	26.9 ± 3.1	7,037	2,128
Dallas, TX	2001–5	918	216	34.4 ± 10.6	14.0 ± 6.5	4.4 ± 2.9	3.3 ± 1.6	0.5 ± 0.3	27.3 ± 3.7	32,795	7,702
Houston, TX	2000–5	918	237	25.4 ± 11.2	14.3 ± 6.4	4.1 ± 2.8	2.1 ± 1.6	0.5 ± 0.2	27.6 ± 2.6	52,284	13,197
Beaumont, TX	2001–5	918	210	35.7 ± 7.2	9.2 ± 3.8	1.6 ± 1.0	2.5 ± 1.0	0.2 ± 0.1	27.2 ± 2.4	9,704	2,267
Kansas City, MO	2002–4	765	118	38.3 ± 10.2	12.1 ± 5.9	3.5 ± 2.8	2.1 ± 1.2	0.7 ± 0.6	22.7 ± 4.8	12,961	1,843
St. Louis, MO	2000–4	765	241	28.6 ± 9.2	15.6 ± 7.9	5.4 ± 4.1	4.3 ± 1.7	1.0 ± 0.8	23.5 ± 4.5	29,592	9,174
Detroit, MI	2001–3	611	138	27.4 ± 10.6	16.1 ± 9.3	5.3 ± 4.7	3.9 ± 2.0	1.8 ± 1.7	19.9 ± 4.8	27,868	6,305
Cleveland, OH	2001–3	612	118	31.6 ± 11.4	18.0 ± 9.7	6.8 ± 5.0	4.5 ± 2.1	1.8 ± 1.6	19.6 ± 4.7	23,363	4,171
Pittsburgh, PA	2001–3	612	138	32.1 ± 10.5	18.4 ± 10.2	8.8 ± 6.6	4.5 ± 2.2	0.9 ± 0.6	19.7 ± 4.4	26,331	5,989
Buffalo, NY	2002–5	915	81	35.9 ± 13.1	15.2 ± 9.2	6.1 ± 5.2	3.0 ± 1.4	1.2 ± 1.0	18.7 ± 4.7	22,921	1,878
Rochester, NY	2001–5	896	120	30.6 ± 11.9	12.6 ± 8.3	4.8 ± 4.2	2.3 ± 1.5	0.9 ± 0.8	18.7 ± 4.7	15,160	1,995
Philadelphia, PA	2001–3	612	112	29.7 ± 11.7	15.8 ± 10.4	6.0 ± 5.4	3.9 ± 2.0	1.4 ± 1.2	22.4 ± 4.5	25,535	4,548
Boston, MA	2000–4	765	155	27.7 ± 11.2	13.1 ± 7.5	3.8 ± 3.5	3.7 ± 1.6	0.9 ± 0.7	19.7 ± 4.7	14,210	2,627

temp, temperature. Values are mean ± SD except where noted otherwise.

a Communities are sorted from west to east and are named after the major city in the community.

b Only data from May–September are used each year; for these years, all data available (mortality, ozone, PM_2.5_ mass, and PM_2.5_ speciation).

c Community average.

**Table 2:** Sensitivity analysis of temperature and dew point temperature on ozone–mortality effect estimate.

Temperature parameterization	Ozone effect (95% CI)
Linear 3-day running mean temp and dew point temp (original)	0.89 (0.45–1.33)
Cubic regression splines (2) with 3 df running mean temp and dew point temp	0.93 (0.50–1.35)
Linear 3-day running mean temp and dew point temp; unaveraged temp and dew point temp	0.79 (0.40–1.18)
Cubic regression splines (4) with 3 df 3-day running mean temp and dew point temp; unaveraged temp and dew point temp	0.80 (0.40–1.21)
Removal of hottest 2% of days in each community. Reanalysis with linear 3-day running mean temp and dew point temp	0.78 (0.26–1.29)
Linear same-day temp and dew point temp	0.48 (0.05–0.90)
Quadratic terms for same-day temp and dew point temp (centered)	0.45 (0.004–0.89)

temp, temperature. Ozone effect is represented as percent increase in nonaccidental mortality with 10-ppb increase in same-day 24-hr summertime ozone concentration.

**Table 3:** Matrix of Pearson correlation coefficients between all pollutants examined.

Pollutant	Ozone	PM_2.5_	Sulfate	OC	Nitrate
Ozone	1	0.43	0.34	0.50	0.24
PM_2.5_	0.43	1	0.86	0.64	0.48
Sulfate	0.34	0.86	1	0.45	0.15
OC	0.50	0.64	0.45	1	0.38
Nitrate	0.24	0.48	0.15	0.38	1

**Table 4:** Percent increase in nonaccidental mortality per 10-ppb increase in same-day 24-hr summertime ozone concentration adjusted for PM_2.5_ total mass, sulfate, OC, and nitrate.

	Percent of ozone days	Ozone effect (95% CI)	Adjusted ozone effect (95% CI)
Ozone	100	0.89 (0.45 to 1.33)	—
PM_2.5_	84	0.88 (0.39 to 1.36)	0.79 (0.27 to 1.31)
PM_2.5_ cubic regression spline	84	0.88 (0.39 to 1.36)	0.82 (0.25 to 1.38)
Sulfate	18	0.85 (–0.01 to 1.55)	0.58 (–0.33 to 1.49)
OC	17	0.51 (–0.36 to 1.36)	0.54 (–0.36 to 1.45)
Nitrate	17	0.74 (–0.10 to 1.58)	0.62 (–0.21 to 1.45)
